# Combined skin dilator and titanium mesh application to repair scalp and skull defects: a case report

**DOI:** 10.1093/jscr/rjae148

**Published:** 2024-03-15

**Authors:** Chao Li, Wen Nie, Fujun Wang

**Affiliations:** Department of Burn and Plastic Surgery, Heze Municipal Hospital, No 2888 Caozhou Street, Shandong Province, Heze, China; Department of Neurosurgery, Jiaozhou Hospital of Tongji University Dongfang Hospital, No 88 Guangzhou street, Shandong Province, Qingdao, China; Department of Urology, Heze Municipal Hospital, No 2888 Caozhou Street, Shandong Province, Heze, China

**Keywords:** high-voltage electrical burns, scalp and skull defects, skin dilator, titanium mesh

## Abstract

We report a case of skin dilator combined with titanium mesh to repair scalp and skull defects. An 18-year-old male with a scalp defect and skull necrosis due to high-voltage electrical burns was admitted to our hospital. In the first stage, the wound was debrided, antibiotics were applied to control the infection, and two skin dilators were embedded under the scalp after debridement. In the second stage, necrotic skull material was removed, the skull defect was repaired using titanium mesh, and the scalp defect was repaired by transferring expanded flaps. The patient was followed up for 6 months, recovering well and achieving a satisfactory head shape.

## Introduction

Skin dilators play an important role in repairing skin defects. In the 1950s, the American plastic surgeon Neumann first used skin dilators for auricular reconstruction, while Chinese plastic surgeons began to use skin dilators in 1984 [1]. Titanium alloy has the features of inertia and good histocompatibility, and titanium mesh was a common and popularly used material for cranioplasty to restore cranial integrity [2]. We report the case of a young man with a scalp defect and skull necrosis due to high-voltage electrical burns; in this case, skin dilators combined with titanium mesh were applied to repair the scalp and skull defects.

## Case report

An 18-year-old male with a scalp defect and skull necrosis due to high-voltage electrical burns was admitted to our hospital. Physical examination found a defect of the patient’s scalp of ~4 cm × 5 cm on the top of the head on the right side and the surrounding tissue of the defect was scorched, with part of the exposed skull being blackened. The patient was treated by debridement and antibiotics.

**Figure 1:**
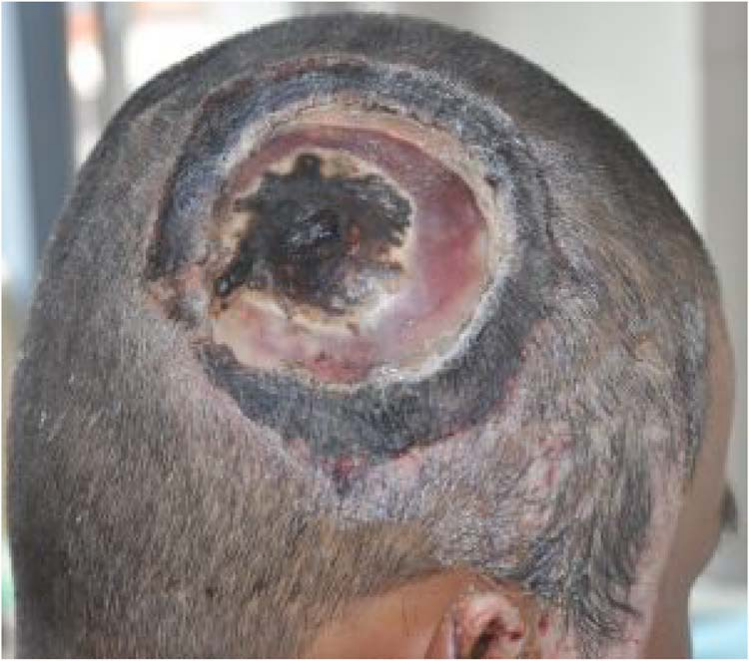
Skull exposure of ~8 cm × 9 cm, the bone being dark, and a cortex skull defect of ~3 cm × 3 cm.

**Figure 2:**
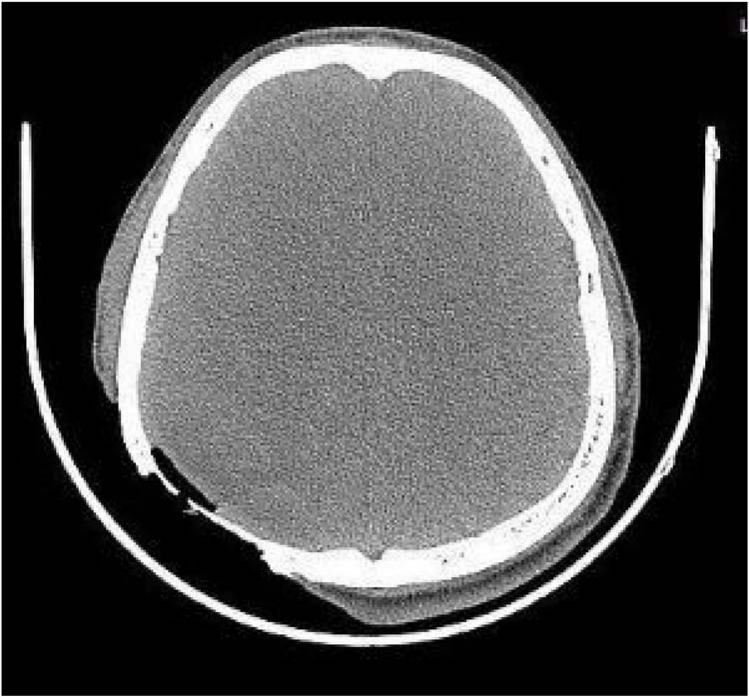
CT shows the presence of gas inside the skull.

**Figure 3:**
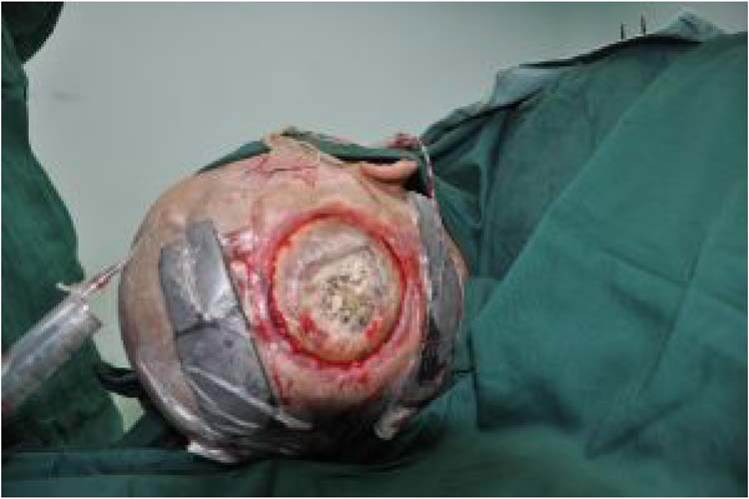
Defect of the inner plate of the skull of ~1 cm × 1 cm, with exposure of the dura mater.

**Figure 4:**
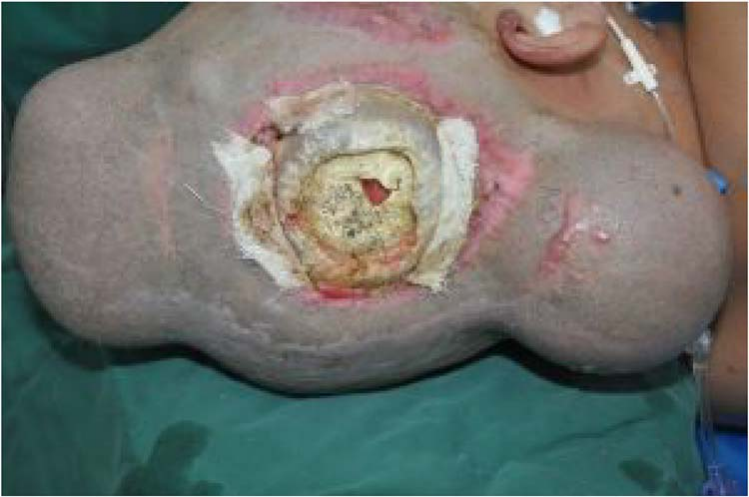
Regular injection of two dilators for dilation.

On post-debridement Day 16, the exposed skull was ~8 cm × 9 cm, the bone was dark, and the cortex of the skull defect was ~3 cm × 3 cm (Fig. 1). Computed tomography showed the presence of gas inside the skull (Fig. 2). Bacterial culture of wound secretions reported *Proteus mirabilis*. During residual wound debridement, a defect of ~1 cm × 1 cm of the inner plate of the skull and dura mater exposure were found (Fig. 3). Subsequently, two skin dilators were embedded under normal scalp and were dilated by regularly injecting saline (Fig. 4). The exposed dura was regularly rinsed with saline to reduce bacterial colonization.

**Figure 5:**
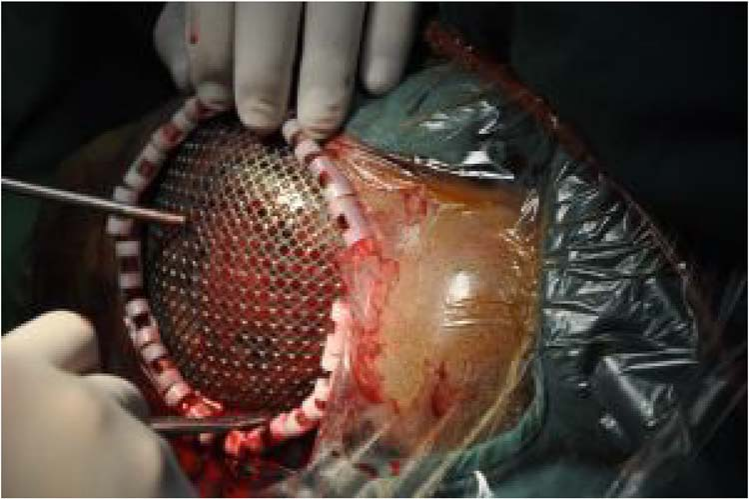
Skull defect repair using titanium mesh.

**Figure 6:**
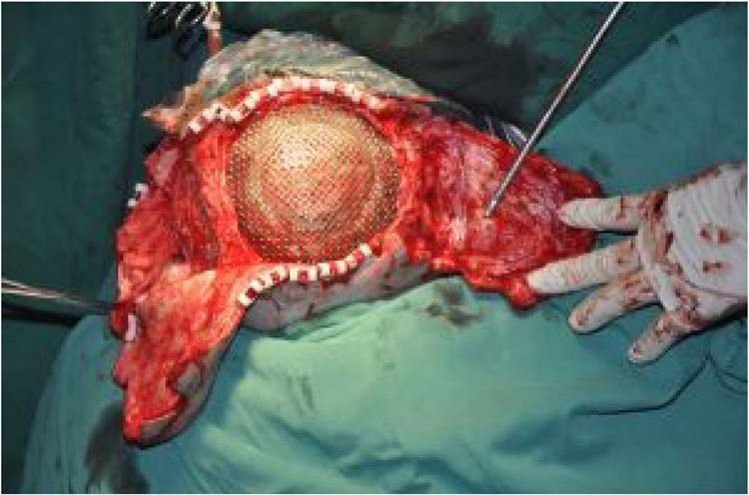
Transfer of two dilated skin flaps to repair the scalp defect.

On post-debridement Day 85, the necrotic skull was removed by a neurosurgeon and the skull defect was repaired with titanium mesh (Fig. 5). Additionally, the two skin dilators were removed and two expanded skin flaps were transferred to repair the scalp defect (Fig. 6). After 8 days, the incision had healed well and the patient was discharged.

**Figure 7:**
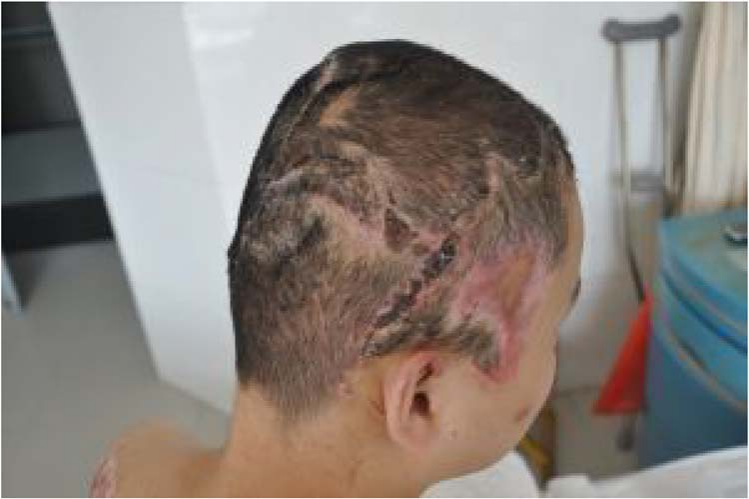
Good survival of the two skin flaps 1 month after discharge.

**Figure 8:**
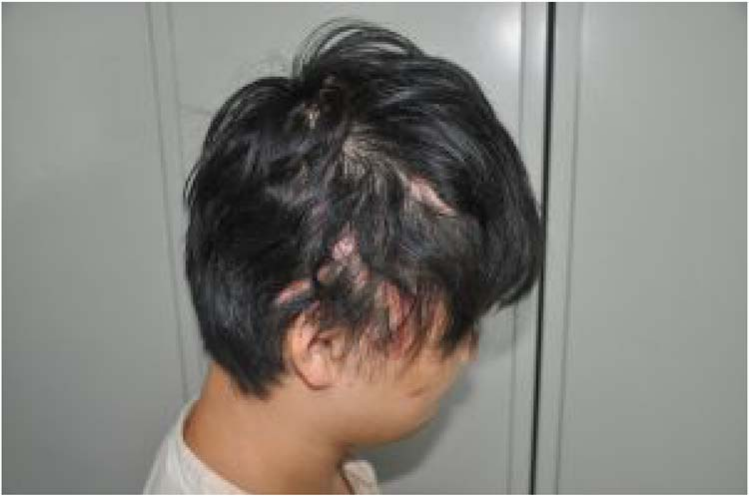
Normal hair growth without alopecia at 6 months after discharge.

The patient was followed up after 1 month, and the two skin flaps had survived well (Fig. 7). At the 6-month follow-up, the hair had grown normally without alopecia (Fig. 8).

## Discussion

High-voltage electrical contact with the head can cause scalp burn and skull necrosis. For wounds with large scalp defects combined with full-thickness skull necrosis, traditional methods of treating such wounds are as follows: local, pedicled, and free flaps were used to repair the wound in the first stage, with dilators subsequently embedded, and the skull and bald hair were repaired in the second stage [3, 4]. The above methods require a long treatment process and many scars will remain in the donor area. There is a greater risk of infection during skull repair when using dilators, and for patients with skull defects with infection, more caution is needed before titanium mesh application. Staged reconstruction should be considered in patients with a large scalp defect and severe soft tissue infection [5]. In the present case, the treatment plans were made through multidisciplinary collaboration. Local infection was controlled by debridement and antibiotics, two skin dilators were embedded and regularly dilated, the exposed dura was regularly rinsed with saline, and, finally, single-stage repair of the skull and scalp defects was performed with titanium mesh and expanded flaps, respectively. Garst *et al.* [6] considered single-stage titanium mesh cranioplasty to be a safe option for the treatment of depressed traumatic skull fractures when autologous repair was not possible. Our treatment measures shortened hospitalization duration and reduced hospitalization frequency and costs.

In conclusion, in the case of dural exposure, skin dilators were used to dilate the skin flaps, and, finally, the head defect was repaired with titanium mesh and expanded flaps by a single-stage surgical operation, which has rarely been reported in the literature.
